# Motor fluctuations due to interaction between dietary protein and levodopa in Parkinson’s disease

**DOI:** 10.1186/s40734-016-0036-9

**Published:** 2016-05-26

**Authors:** Tuhin Virmani, Sirinan Tazan, Pietro Mazzoni, Blair Ford, Paul E. Greene

**Affiliations:** Department of Neurology, College of Physicians and Surgeons, Columbia University, New York, NY USA; Current addresses: University of Arkansas for Medical Sciences, 4301 W. Markham St., #500, Little Rock, AR 72205 USA; Healthcare Partners, 3565 Del Amo Blvd., Ste 200, Torrance, CA 90503 USA; Mt. Sinai School of Medicine, Box 1637, New York, NY 10029 USA

**Keywords:** Protein effect, Levodopa, Motor fluctuations, Parkinson disease

## Abstract

**Background:**

The modulation of levodopa transport across the blood brain barrier by large neutral amino acids is well documented. Protein limitation and protein redistribution diets may improve motor fluctuations in patients with Parkinson’s disease but the pharmacokinetics and pharmacodynamics of levodopa and amino acids are highly variable.

**Methods:**

Clinical records of 1037 Parkinson’s disease patients were analyzed to determine the proportion of patients with motor fluctuations related to protein interaction with levodopa. Motor fluctuations due to protein interaction with levodopa were defined as dietary protein being associated with (i) longer time to levodopa effectiveness, (ii) reduced benefit or duration of benefit, (iii) dose failures or (iv) earlier wearing off from a previously effective dose. Dose failures, sudden, painful or behavioral wearing-off periods, gait freezing, nausea, hallucinations, orthostasis, and dyskinesias were taken as markers of motor fluctuations, disease severity, and levodopa side effects potentially influenced by protein.

**Results:**

5.9 % of Parkinson’s disease patients on levodopa, and 12.4 % with motor fluctuations on levodopa correlated their fluctuations with the relative timing of levodopa and protein intake. These patients were younger at disease onset, had worse motor fluctuations and had a higher incidence of family members with Parkinson’s disease. Early wearing off or decreased dose efficacy were most commonly associated with protein interaction. 60 % of patients who modified their diets had weight loss.

**Conclusions:**

This study suggests that clinically significant protein interaction with levodopa may occur mostly in a subset of Parkinson’s disease patients with earlier disease onset and those with familial disease.

## Background

The reduction of the response to levodopa by amino acids [1] and dietary protein [2] was first noted by Cotzias and colleagues. Dietary modifications have been tested in Parkinson’s disease (PD) patients with motor fluctuations. These include low [2–5] and high protein diets [6, 7], addition of dietary large neutral amino acids (LNAAs) [1, 8, 9] and redistribution of daily protein intake [3, 7, 10–16] (the majority of dietary protein is normally consumed with dinner). The improvement in clinical response varied from 30 % with protein redistribution [11] to 82 % with low-protein diets [5]. The ability of patients to maintain such diets long-term was variable [13, 16].

LNAAs (e.g. phenylalanine, tyrosine, and tryptophan) have been shown to compete with levodopa for absorption into the brain [17–20]. However, the data supporting a role for plasma LNAA concentrations in motor fluctuations is variable. Mean plasma levodopa levels were unchanged with low protein and protein distribution diets compared to a regular diet [3], but paradoxically higher on high protein diets [6, 7]. Peak plasma levodopa concentrations were similarly variable [4, 6, 7, 21, 22]. While levels of LNAAs are lower after meals with lower protein [3, 6, 7], the fluctuation in LNAAs with regular hospital meals was small compared to the large swings in plasma levodopa levels [9].

These studies were conducted on small populations with heterogeneous data making it difficult to recommend dietary modification to all PD patients [23]. In this study we aimed to establish the prevalence and characteristics of PD patients with clinically significant protein interaction with levodopa leading to motor fluctuations.

## Methods

Of all the patients seen at the Columbia University Movement Disorders center between 2000 and 2012 by P.M., B.F. and P.E.G., with available clinical records, a total of 1037 patients with a clinical diagnosis of idiopathic PD by UK Brain bank criteria [24] were seen, and their electronic clinical notes were reviewed and the parameters described below were extracted and tabulated. Eight hundred seventy seven patients were on levodopa and 435 of these had multiple clinic visits. All available visit notes were reviewed and used to determine the presence, and in a subset, the onset of the symptoms as outlined for each parameter below.

Motor fluctuations were considered present if levodopa dosing was reported by the patient to last less than every 4 hours due to wearing off (“OFF”) of the motor benefit derived from levodopa. Motor fluctuations related to protein interaction with levodopa (PIL) were considered present when meals with higher protein food groups (meat, eggs, dairy predominantly), were reported by the patient to be followed by any of (i) longer time to levodopa effectiveness, (ii) reduced benefit or duration of benefit, (iii) dose failures or (iv) earlier wearing off from a previously effective dose. Since patients may inconsistently report protein ingestion effecting levodopa effectiveness, and although all three movement disorders neurologists typically ask about protein interaction in patients with motor fluctuations, this may be an underestimate. Additionally, if patients reported that meals in general resulted in decreased effectiveness of levodopa, but did not specifically correlate this to the presence of high protein content, they were not considered to have PIL for the purpose of this study. This was done in order to try and only address protein/amino acid effects related to competing transportation with levodopa across the blood brain barrier and exclude potential issues related to any large meal leading to changes in gastric emptying and thereby altering levodopa absorption from the gut.

The age at motor onset was determined based on the year patients reported onset of their motor symptoms subtracted from their year of birth. The duration of disease was determined from the year of motor symptom onset and the year of the last (i.e. most recent) clinic note that was reviewed. The presence of freezing of gait (as a marker of disease severity) was determined based on patient report in the clinical history of the sensation of the feet sticking to the ground on initiation of gait, turning, in tight spaces or at destination or documented by the movement disorders neurologist on examination. Levodopa side effects (as potential markers influenced by PIL) of nausea, hallucinations, and orthostasis (symptomatic lightheadedness on standing) were deemed present if documented in the clinical history and dyskinesias present if documented in the clinical history or on movement disorders examination.

The year patients started levodopa therapy was available for 37/52 patients with PIL and 487/825 patients without PIL, and was used to calculate the years on levodopa based on the last recorded clinic note. In 15/52 patients the year they noted protein interaction led to motor fluctuations was documented and was used to determine the time of onset of PIL from motor symptom onset and levodopa therapy initiation. The total daily levodopa dose was calculated based on 100 % bioavailability of carbidopa/levodopa immediate release (IR) and carbidopa/levodopa/entacapone formulations [25] and an estimated 70 % bioavailability of carbidopa levodopa extended release (CR) formulation [26]. The presence of concurrent dopamine agonist use (ropinirole, pramipexole, rotigotine, pergolide or bromocriptine) or the use of deep brain stimulator (DBS) surgery for the treatment of their PD was also tabulated from the clinical charts. The report of 1st and/or 2nd degree relatives with PD in the social history was used for the calculation of the percentage with family history in the groups.

In order to exclude significant differences in age of onset or disease duration in the PIL vs no-PIL groups as a cause for the changes we observed, two subgroups of no-PIL patients were evaluated. The no-PIL group was sorted by age of onset and for each patient in the PIL group, five patients in the no-PIL group with the same age of onset were randomly included in the age of onset subgroup (no-PIL: age-ons). In the few cases where enough patients with the exact age of onset were not available, patients above and below that age were included, keeping the mean age of onset as close as possible to that of the patient with PIL. This subset of patients was then analyzed and compared to the PIL group for the other parameters described above. In a similar manner a subgroup of patients was also produced that included five randomly selected patients without PIL with the same disease duration as each patient with PIL (no-PIL: dis-dur).

The Shapiro-Wilk test was applied for data normality and statistical significance determined by chi-square or Mann–Whitney test where appropriate. SPSS version 22 (IBM) was used for statistical analysis and box plots. The study was approved by the Columbia University Institutional Review Board.

## Results

**Fig. 1:**
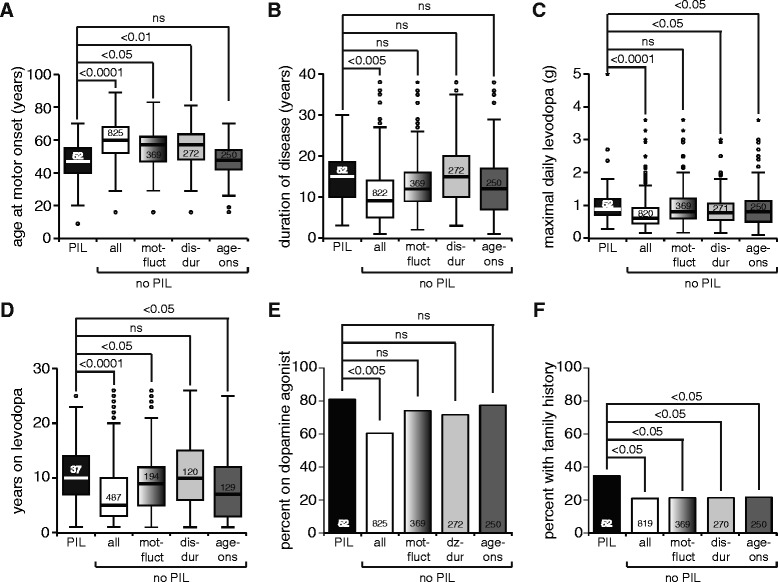
General characteristics of all patients who exhibited protein interaction with levodopa **a** Age at motor onset, **b** duration of disease, **c** maximal daily levodopa dose, **d** years on levodopa, **e** percentage of patients on a dopamine agonist and **f** percentage with first or second degree relative with parkinsonism. (PIL; *black bars*) compared to those that did not (no-PIL: all; *white bars*) and subgroups of PIL patients with motor fluctuations (no-PIL:mot-fluct; *gradient bars*) and no-PIL patients matched for disease-duration (no-PIL: dis-dur; *light gray bars*) and age-at-motor-onset (no-PIL: age-ons; *dark gray bars*). The numbers of patients represented in each graph are inset. *P* values represent results of the chi-square or Mann–Whitney test comparing each no-PIL group to the PIL group. Legend: PIL: protein interaction with levodopa, no-PIL: no protein interaction with levodopa

**Fig. 2:**
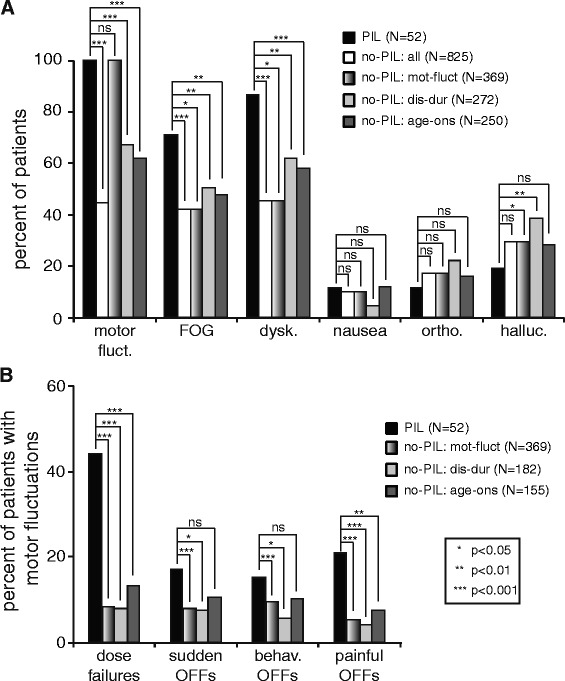
**a** Motor and non-motor characteristics of all patients (*black bars*: PIL, *white bars*: no-PIL: all) and subgroups of no-PIL patients with motor fluctuations (no-PIL: mot-fluct; *gradient bars*), or matched for disease-duration (no-PIL:dis-dur; *light gray bars*) or age-at-motor-onset (no-PIL: age-ons; *dark gray bars*). **b** Characteristics of motor fluctuations in PIL patients compared to those with motor fluctuations in the no-PIL groups. *P* values represent results of the chi-square or Mann–Whitney test comparing each no-PIL group to the PIL group. Legend: PIL: protein interaction with levodopa, no-PIL: no protein interaction with levodopa, motor fluct: motor fluctuations, behav. OFFs: behavioral OFFs, FOG: freezing of gait, dysk: dyskinesias, ortho.:orthostasis, halluc.: hallucinations

Of the 1037 PD patients, 877 took levodopa, and 52/877 (5.9 %) met criteria for motor fluctuations related to PIL. Patients reporting PIL were younger at motor symptom onset, took higher maximal daily equivalent levodopa doses, used more dopamine agonists, had longer disease duration and levodopa use (Fig. 1a–e), and were younger at their last clinic visit (62.2 ± 10.8 vs. 68.8 ± 10.5 years). Gender ratio was similar in both groups (37 vs. 31 % female PIL vs. no-PIL patients). The percentage of patients who had undergone DBS was also similar in both groups (7.7 vs. 8.5 % PIL vs. no-PIL patients). PIL patients reported more family members (first and/or second degree relatives) with PD (*p* = 0.024; OR = 1.96; 95 % CI = 1.08–3.56) (Fig. 1f). More PIL patients had dyskinesias and freezing of gait, but there was no difference in nausea, orthostasis or hallucinations (Fig. 2a).

Of the 52 patients with PIL, 26 (50 %) reported decreased efficacy of levodopa after protein intake, 15 (29 %) reported that protein intake led to wearing OFF of a previously effective dose, 9 (17 %) reported dose failures when levodopa and protein were taken concurrently, while one patient each reported delayed time to motor improvement (ON state) and decreased length of ON state time after protein. In PIL patients with data, PIL onset was 12.9 ± 6.7 years after motor onset (range 3–26 years, *N* = 15) and 7.9 ± 7.7 years after starting levodopa (range 0–25 years, *N* = 8).

Twenty patients had documented dietary modifications ranging from decreased total daily protein intake (15/20), redistribution of protein to the evening meal (2/20), small frequent meals (1/20), decreased total protein all taken only with the evening meal (1/20), and small frequent meals with protein in the evening meal only (1/20). Two patients had not changed their diets. The efficacy of these changes on motor fluctuations was unfortunately not adequately documented but 12/20 (60 %) reported weight loss after changing their diet.

In PD patients with motor fluctuations, a population much less likely to suffer from potential underreporting, 52/421 (12.4 %) had PIL, with a greater frequency of dose failures, sudden OFFs, behavioral OFFs and painful OFFs compared to no-PIL patients with motor fluctuations (Fig. 2b). Hallucinations were less common in PIL patients compared to no-PIL patients with motor fluctuations (Fig. 2a). The duration of disease from time of onset of motor symptoms (Fig. 1b) and the maximal daily levodopa dose (Fig. 1c) were not statistically different between these groups. However, the duration of levodopa use was longer in PIL patients (Fig. 1d). The age at motor onset was earlier (Fig. 1a) and the percent reporting a family history of PD remained higher in PIL patients (*p* = 0.037; OR = 1.92; 95 % CI = 1.03–3.59) (Fig. 1f).

To further examine the longer disease duration in PIL patients, we performed a subgroup analysis by randomly selecting, for every PIL patient, five no-PIL patients matched for disease duration. In this subgroup (no-PIL: dis-dur), age at motor onset was again significantly lower in PIL patients (Fig. 1a), while duration of levodopa use (Fig. 1d) and percentage on dopamine agonists (Fig. 1e) were equivalent. Similarly to the whole-group results, PIL patients in this subgroup took a higher levodopa dose (Fig. 1c) and had higher frequency of motor complications (Fig. 2) and family history of PD (*p* = 0.041; OR = 1.94; 95 % CI = 1.02–3.67) (Fig. 1f). Therefore, differences in disease duration are unlikely to account for the differences between PIL and no-PIL groups.

Younger-onset patients are known to have a higher incidence of familial disease [27–29]. To determine whether there was a familial component to PIL independent of age, we matched PIL patients for age of onset (Figs. 1 and 2; no-PIL: age-ons). Similar to the whole-group comparison, the percentage of patients with PIL reporting a family history of PD was higher (*p* = 0.045; OR = 1.92; 95 % CI = 1.01–3.67) (Fig. 1f).

## Discussion

A significant protein interaction with levodopa associated with motor fluctuations was reported in a small percentage of our patients: 5.9 % of our PD patients on levodopa and 12.4 % of those on levodopa with motor fluctuations. Almost 80 % of PIL patients either had decreased efficacy of their levodopa dose, or wore OFF from a prior dose after protein ingestion. PIL patients were younger at disease onset and reported a higher frequency of familial PD, even when matched for disease duration, maximal daily levodopa dose, or years of levodopa use, suggesting that PIL could be familial. The higher frequency of familial disease could not be accounted for by an earlier age of onset alone.

PIL patients also had more severe motor fluctuations with more frequent dose failures, sudden OFFs, behavioral OFFs and painful OFFs compared to no-PIL patients with motor fluctuations, which could not be accounted for by longer disease duration in the PIL group. The severity of motor fluctuations could be due to the interaction with levodopa reducing its effectiveness and therefore leading to worsened signs and symptoms of the disease. However it could potentially be a manifestation of differences in the disease process itself, as PIL patients also had a greater frequency of freezing of gait when compared with all no-PIL patients including matched for disease duration and age of onset subgroups of no-PIL patients analyzed. Additionally the mean onset of PIL from onset of motor symptoms was 12.9 years in the subset for which data was available, and the mean duration of disease in the no-PIL group with motor fluctuations was the same suggesting that they had a long enough disease duration to develop such complications were they to occur.

In the subset of patients with both the time of levodopa initiation and the time of onset of PIL available, on average 8 years elapsed between levodopa initiation and PIL development. This suggests that allowing patients to take levodopa with meals initially, to avoid developing nausea, should not result in a significantly decreased benefit. Additionally, limiting protein in the diet or ingesting protein primarily with the evening meal led to weight loss in a majority of PIL patients. As patients with PD are already at an increased risk for weight loss [30], limiting their diet can be problematic. Apomorphine may be a better alternative to dietary protein restriction or redistribution, unless it cannot be tolerated due to nausea.

This study has the limitations of a retrospective study. We might underestimate the prevalence of PIL due to underreporting by patients or due to physicians not consistently asking about protein-levodopa effects. However the three fellowship trained movement disorders neurologist routinely ask about protein related motor OFF states, making the 12.4 % reported PIL in PD patients with motor fluctuations, less likely to be an underestimate, if at all. As patients often are not aware of the pattern of their motor fluctuations in relation to medications they may not notice the effect of meals or protein on their motor function, and therefore not report it. However the opposite is also not uncommon, whereby patients only take levodopa on an empty stomach as they are advised by their physicians or pharmacists of an interaction with protein. While this could be a significant factor in reporting by patients early in the disease course before onset of motor fluctuations, experienced patients with over 10 years disease duration, patients experiencing motor fluctuations, undergoing care at a tertiary referral center, and having their medications adjusted based upon their reports of motor fluctuations, are likely to be more aware of factors that can precipitate or worsen their motor function. In a population with over 10 years of disease, the incidence of DBS surgery was similar in both groups making it also unlikely to account for no-PIL patients not noting protein interaction due to less severe motor fluctuations from DBS therapy.

In clinical practice medications are always being adjusted based on patient’s subjective reports of their motor function with levodopa dosing and would be a factor whether this were a prospective or retrospective study. While most patients reported high protein meals causing the effect and not food in general (these patients were excluded from the PIL group), we cannot account for the effect delayed gastric emptying may potentially have had in the motor response in these patients or the role fat content of meals may play in this process. However reduced benefit from a dose, earlier wearing off from a previously effective dose and decreased duration of benefit from a dose are less likely to be due to delayed gastric emptying and accounted for the majority of patients effects (79 %) in this cohort. Calculations of duration of levodopa use, time to PIL onset from motor symptom onset or levodopa initiation was limited to a subset of patients. Additionally the efficacy of dietary modifications could not be determined from records available. However, the large numbers of patient records analyzed (over 1000) and the availability of serial office notes in about 50 % of these patients helps minimize some of these concerns.

## Conclusion

While it has been suggested that levodopa can be reduced in patients ingesting less protein [2, 5, 16], patients not responding to protein redistribution diets were younger at onset and had longer duration of levodopa use [16], as were our patients with PIL. This finding, together with the unclear mechanism, the small percentage of patients reporting PIL in our study, and the weight loss experienced by those changing their diets, raises the question as to whether dietary modification should be recommended to all patients reporting motor fluctuations, or as some suggest, to all patients taking levodopa.

## Abbreviations

LNAA: large neutral amino acids
PD: Parkinson disease
PIL: protein interaction with levodopa
